# Plasma Metabolic Profiling of Pediatric Sepsis in a Chinese Cohort

**DOI:** 10.3389/fcell.2021.643979

**Published:** 2021-02-15

**Authors:** Guo-Bang Li, Hong-Rong Hu, Wen-Feng Pan, Bo Li, Zhi-Ying Ou, Hui-Ying Liang, Cong Li

**Affiliations:** ^1^Department of Biochemistry, Zhongshan School of Medicine, Sun Yat-sen University, Guangzhou, China; ^2^Central Laboratory, Affiliated Dongguan People’s Hospital, Southern Medical University, Guangzhou, China; ^3^Department of Neurosurgery/Neuro-oncology, Sun Yat-sen University Cancer Center, State Key Laboratory of Oncology in South China, Collaborative Innovation Center for Cancer Medicine, Guangzhou, China; ^4^Affiliated Guangzhou Women and Children’s Hospital, Zhongshan School of Medicine, Sun Yat-sen University, Guangzhou, China

**Keywords:** sepsis, metabolomics, amino acids, fatty acids, carbohydrates

## Abstract

Sepsis represents one of the most pressing problems in pediatrics, characterized by pathogenic bacteria invading the blood, growing and multiplying in the blood circulation, and ultimately causing severe infections. Most children with sepsis have a rapid disease onset and frequently exhibit sudden high fever or first chills. Here we performed comprehensive metabolomic profiling of plasma samples collected from pediatric sepsis patients to identify specific metabolic alterations associated with these patients (*n* = 84, designated as case subjects) as compared to healthy cohorts (*n* = 59, designated as control subjects). Diagnostic models were constructed using MetaboAnalyst, R packages, and multiple statistical methods, such as orthogonal partial least squares-discriminant analysis, principal component analysis, volcano plotting, and one-way ANOVA. Our study revealed a panel of metabolites responsible for the discrimination between case and control subjects with a high predictive value of prognosis. Moreover, significantly altered metabolites in sepsis survivors versus deceased patients (non-survivors) were identified as those involved in amino acids, fatty acids, and carbohydrates metabolism. Nine metabolites including organic acids and fatty acids were also identified with significantly higher abundance in sepsis patients with related microbes, implicating greater potentials to distinguish bacterial species using metabolomic analysis than blood culture. Pathway enrichment analysis further revealed that fatty acid metabolism might play an important role in the pathogenesis of sepsis.

## Introduction

Sepsis is a complication of infectious processes associated with substantial morbidity and mortality, especially in childhood patients (Kaukonen et al., 2014). The incidence of sepsis varies by different social-economic regions, and it remains a global health concern. There are more than 18 million severe sepsis cases worldwide each year (Kumar et al., 2011; Fleischmann-Struzek et al., 2018). In the United States, sepsis and septic shock affect up to 300 patients per 100,000 people per year, and the annual number is increasing at a rate of 1.5 to 8.0% (Torio and Moore, 2006). According to epidemiological surveys, sepsis is one of the leading causes of death in the intensive care unit (ICU), and there is an urgent need to improve the diagnosis and treatment of severe sepsis patients (Angus and Van der Poll, 2013). Sepsis is defined as patients who are acutely infected and demonstrate two or more systemic inflammatory response syndromes (SIRS), including temperature >38°C or <36°C, heart rate >90/min, respiratory rate >20/min or PaCO_2_ <32 mm Hg (4.3 kPa), and white blood cell count >12,000/mm^3^ or < 4000/mm^3^ or >10% immature bands (Singer et al., 2016). The key pathological components to develop sepsis is the runaway inflammatory responses to infection, and it is postulated that sepsis could be triggered by numerous metabolic products, such as proinflammatory mediators responsive to infectious agents, alien products generated by invading bacteria, and those released by damaged cells (Bosmann and Ward, 2013; Drosatos et al., 2015). It is noted that sepsis is caused by infection, but sepsis itself is not contagious; therefore, by nature, sepsis is the body’s response to infectious factors.

Blood culture is the gold standard for sepsis diagnosis. However, only one-third case is commonly tested positive by blood culture, and approximately 30% sepsis samples from multiple sites of one individual patient are tested negative (Opal et al., 2003; Ranieri et al., 2012). Patients with sepsis have a certain disturbance of whole-body metabolism during the early stage of infection, and the dynamic changes of the metabolic network can be detected by metabolomic analysis to provide specific diagnostic markers, microbiota classification, and prognosis prediction (Langley et al., 2013; Fischer et al., 2014; Wang et al., 2016). According to recent research data, energy metabolism, including glycolysis, gluconeogenesis, and lipid metabolism, is elevated in patient serum with sepsis. However, there is no clear serum biomarker for distinguishing different sources of infection (such as Gram-negative or Gram-positive bacteria). In terms of prognostic judgment, it mainly depends on clinical indicators, which do not always reflect disease severity. Therefore, identifying metabolic biomarkers with great predictive power will be of great significance for the diagnosis and treatment of patients with sepsis.

Metabolomics is a technique quantitatively analyzing all metabolites involved in certain pathological processes, and the resultant metabolic landscape can be used to improve the understanding of disease pathogenesis including sepsis (Guo et al., 2015). In this study, we aim to characterize the plasma metabolic signatures in sepsis survivors, non-survivors, and control subjects, by employing high-performance liquid chromatography and mass spectroscopy (HPLC-MS)-based metabolomics. These data set a solid foundation for constructing an accurate metabolic model for predicting sepsis patient outcome.

## Materials and Methods

### Clinical Subjects

The peripheral blood samples of all participants in this retrospective study were collected from Guangzhou Women and Children’s Medical Center in China. Eighty-four pediatric sepsis patients including 52 males and 32 females were admitted to the ICU from 2015 to 2016, and inclusion of the patients was determined by a consensus panel of pediatric and ICU physicians according to the presentation of inflammation and infection, plus hyperthermia or hypothermia (Singer et al., 2016). The extent of infection and infectious origin was adjudicated by polymerase chain reaction after blood culture (Patel et al., 2011; Kirn and Weinstein, 2013). Non-infected controls were healthy children undergoing physical examination in the Guangzhou Women and Children’s Medical Center, with matched age and geographical birthplace. The local ethics committees have approved this study, and parental informed consent was obtained from every participant.

### Statistical Analysis

Continuous clinical variables were presented as the mean ± standard deviation. Metabolic intensities were log2-transformed to normalize non-normal distributions before further analysis. We assessed metabolic profiles of serum samples from sepsis survivors, sepsis non-survivors, and healthy controls by ANOVA and logistic regression by including covariates of sex and age of admittance. MetaboAnalyst and R packages were employed to construct statistical plots, including principal components analysis (PCA) and orthogonal partial least squares-discriminant analysis (OPLS-DA) (Pang et al., 2020). The variable importance in the projection (VIP) value of each variable in the OPLS-DA model was calculated to indicate its contribution to the classification. Metabolites with the VIP value > 1 were further applied to Student’s *t*-test at the univariate level to measure the significance of each metabolite; *q*-values were calculated for multiple testing, with the criteria of VIP > 1 and *p*-value less < 0.05 (Table 2).

### Metabolomic Profiling

High-performance liquid chromatography and mass spectroscopy was performed as described previously (Lawton et al., 2008). Briefly, 10 microliters were taken out from each plasma sample, evenly mixed to generate 20 quality control samples. The mobile phase of positive mode consisted of 0.1% formic acid in water (solvent A) and 0.1% formic acid in acetonitrile (solvent B), and the mobile phase of negative mode consisted of 0.5 mM ammonium acetate in water (solvent A) and 100% acetonitrile (solvent B). Raw MS data were first converted to.mzxml files, then the individual data for each participant were grouped together and processed for further analysis. mzxml files were imported to MetAnalyzer for peak detection, and the peak areas of positive and negative ions were measured according to corresponding internal standard ions. MetDNA was employed to confirm the metabolite identification in untargeted metabolomics (Shen et al., 2019). Quantifications were performed using the weighted linear least squares regression analysis based on fortified calibration standards as prepared before each run. This integrated platform enabled the high-throughput collection and relative quantitative analysis of analytical data and identified a large number and broad spectrum of molecules with high confidence. In this study, a total of 514 small molecules were measured and annotated as metabolites in the positive (281) and negative (233) mode (Wishart et al., 2009).

## Results

### Clinical Characteristics of the Subjects

The general and clinical characteristics of the subjects participating in the study were shown in Table 1. We selected a total of 84 sepsis cases after admission to the ICU with the month of onset ranging from 15 days after birth to 13 years old, and the average onset month was 25.59 ± 35.3. Sepsis cases with any known risk of alternative lethality were excluded. In addition, 59 children with an average age of 3.48 ± 2.43 were recruited from physical examination center as controls. All participants included in our analysis were of Southern Chinese Han origin as reported by their guardian. Sepsis cases could be categorized into two groups based on their final outcome at day 28: sepsis survivors (*n* = 74, 88.1%) and sepsis non-survivors (*n* = 10, 11.9%).

**TABLE 1:** General clinical variables of the sepsis case control cohort.

Characteristics	Sepsis survivors (*n* = 74)	Sepsis death (*n* = 10)	Control (*n* = 59)
Gender (male/female)	39/35	5/5	32/27
Onset month	23.22 ± 35.47	43.1 ± 37.20	NA
Temperature	37.1 ± 0.9	36.8 ± 0.4	NA

*Onset month (mean ± SD): age (month) of onset for sepsis cases.*

### Metabolomic Profiling of Plasma Samples

**FIGURE 1:**
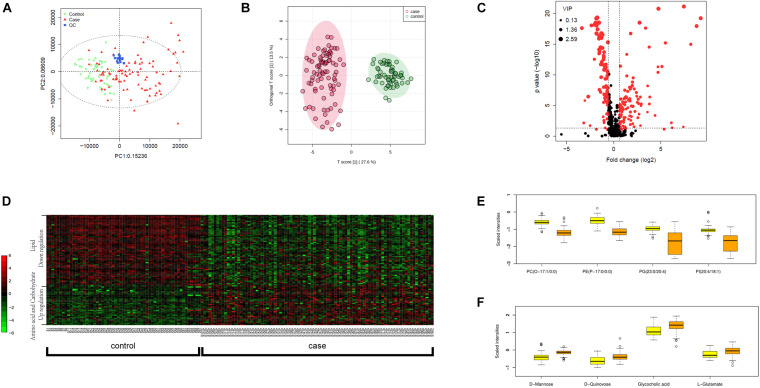
Metabolomic profiling of plasma samples from sepsis cases and control group. **(A)** PCA plot based on the metabolomic analysis. Note that all quality control (QC) samples are clustered. **(B)** OPLS-DA score scatter plots for metabolic profiling of sepsis case (red dots) and control (green dots) group. Discriminations between case and control groups were clearly shown. **(C)** Volcano plot showing significantly altered metabolites in sepsis cases versus controls. Red dots represented metabolites above the thresholds (VIP > 1, *t*-test *P*-value < 0.05, and fold change > 1.5 or < 0.67). **(D)** 521 log-transformed annotated serum metabolites were visualized by a heatmap. Mean intensity was used to indicate the level of differences between case and control samples. **(E)** The four decreased metabolites in sepsis serum presented by scaled intensities in the boxplot (*t*-test *P* < 0.05). **(F)** The four increased metabolites presented by scaled intensities in the boxplot (*t*-test *P* < 0.05).

We carried out metabolic profiling in collected serum samples (separated from peripheral blood specimen) to investigate whether metabolic changes correlate with the severity and prognosis of pediatric sepsis. PCA results showed an ideal aggregation of quality control samples, indicating a stable performance of the metabolomic platform, and the metabolomic profile was obtained with high reproducibility. The orthogonal partial least squares discrimination analysis (OPLS-DA) implicated that the case and control samples were well distinguished by the metabolomic data (Figures 1A,B). The volcano plot highlighted the potential metabolic biomarkers with fold change >1.5 or <0.67, *P*-value < 0.05, and VIP > 1 (Figure 1C). Metabolites with differential abundance were demonstrated by a heatmap plot, as those with increased levels in cases lie in the class of amino acids and carbohydrates, and the decreased panel includes lipids and their derivatives (Figure 1D). In particular, phosphatidylcholine, phosphatidylinositol, and phosphoglyceride were found to be significantly less in cases relative to the control group, and serum levels of several amino acids and carbohydrates were elevated in sepsis serum by over 100% (Figures 1E,F and Table 2).

**TABLE 2:** Metabolites associated with sepsis survivors versus controls, ranked by foldchange.

Metabolites	Class	VIP	Q_value	Foldchange
PE(P-17:0/0:0)	lipid	1.925585	9.14E-19	0.194985
PI(20:4/18:1)	Lipid	1.193777	3.00E-13	0.242166
PC(O-17:1/0:0)	Lipid	2.075808	7.09E-19	0.249678
PA(18:2/7:0)	Lipid	2.165168	4.00E-18	0.30559
Thr-Gly	Oligopeptide	1.48506	6.54E-08	0.382259
PG(23:0/20:4)	Lipid	1.730259	2.12E-12	0.387358
Indoxyl sulfate	Phenol	1.786883	2.56E-10	0.399003
L-Fucose-1-phosphate	Sugar	1.772869	7.94E-13	0.414575
Riboflavin (Vitamin B2)	Acid	0.793711	4.18E-07	14.1998
LPS(20:4/0:0)	Lipid	1.212115	1.56E-09	3.988089
Quinovose	Ketone	1.589411	1.89E-17	3.493425
Glycocholic acid	Acid	1.208684	8.23E-07	3.249042
Mannose	Sugar	1.22824	3.93E-12	1.645824
Glutamate	Amino acid	1.16144	8.17E-05	1.580853

*Q values and VIP are results for the association with sepsis; logistic regression was performed for adjustment of onset month, gender, and liver and kidney diseases status.*

**FIGURE 2:**
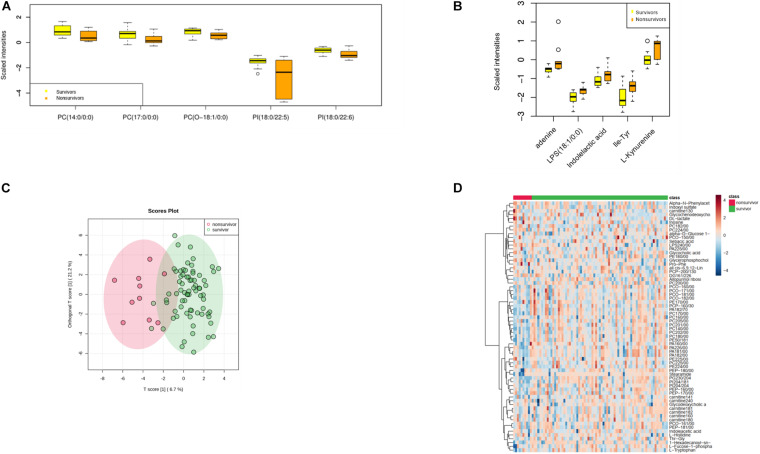
Metabolomic profiling of plasma samples from sepsis survivors and non-survivors. **(A)** The top five decreasing metabolites presented in sepsis non-survivors compared to survivors. The *x*-axis represents specific metabolites, and the *y*-axis shows the normalized scaled intensity. **(B)** The top five increased metabolites in sepsis non-survivors. **(C)** OPLS-DA plot showing the metabolic discrimination between the non-survivor and survivor groups. **(D)** Hierarchical clustering analysis was performed to depict the metabolites that were altered significantly across the non-survivor and survivor groups.

An array of metabolites has been implicated in sepsis-related mortality after logistic regression, with significantly altered abundance between sepsis survivors and non-survivors. Notably, Harmane, Genistein, Metronidazole, Quinolinate, and Cortisone were excluded from further analysis, because these biochemicals were considered to be either drug molecules or products from drug metabolism. The top five decreased metabolites in non-survivors are all lipid species, and the increased level of amino acids and their derivatives was found to be highly correlated with sepsis-related death, including adenine, indolelactic acid, lps(18:1/0:0), Ile-Tyr, and kynurenine (Figures 2A–D).

### Differences in Plasma Metabolites According to Sepsis-Causing Pathogens

**FIGURE 3:**
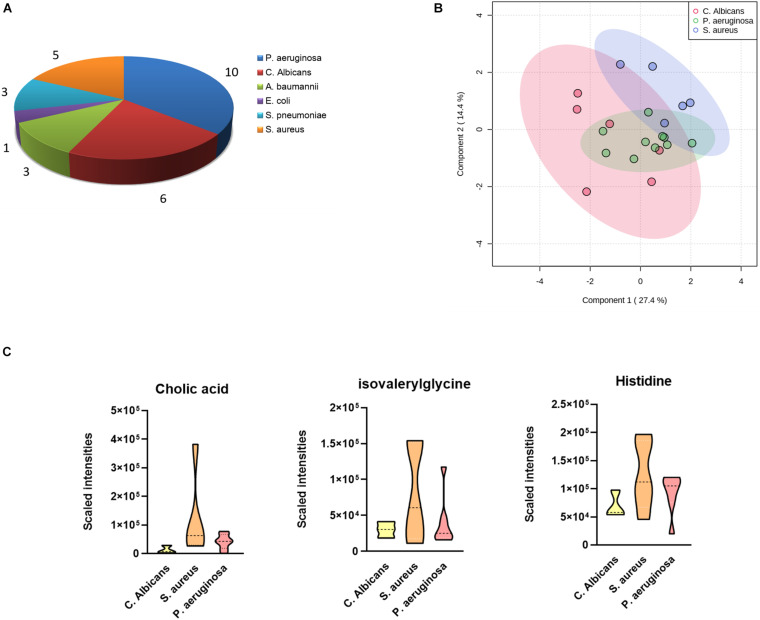
Differences in plasma metabolites according to sepsis-causing pathogens. **(A)** Confirmed sepsis cases infected by different microbes. **(B)** OPLS-DA analysis showing the metabolic discrimination of cases infected by three major microbes: *P. aeruginosa*, *C. albicans*, and *S. aureus*. **(C)** Relative serum levels of cholic acid, isovalerylglycine, and histidine were represented in sepsis survivors infected by *S. aureus*, *P. aeruginosa*, and *C. albicans.*

As the most commonly seen infectious microbes in sepsis cases, *S. aureus, S. pneumoniae*, and *E. coli* contribute to most sepsis cases, especially in children. In our study cohort, only one blood sample was confirmed to be infected by *E. coli*, and the sample was from a 4-year-old girl, cured 63 days after admittance to the hospital. *P. aeruginosa* was the major infective origin (*n* = 10), followed by *C. albicans* (*n* = 6) and *S. aureus* (*n* = 5). We sought to identify differentially expressed metabolites in cases infected by different pathogens. A one-way ANOVA identified nine metabolites including 1-oleoyl-L-alpha-lysophosphatidic acid, cholic acid, hypoxanthine, indoxyl sulfate, isovalerylglycine, histidine, PC(P-16:0/18:1), PI(16:0/18:3), and pregnenolone sulfate with significantly altered abundance in sepsis survivors, non-survivors, and the control group. The serum levels of cholic acid, isovalerylglycine, and histidine were increased in sepsis survivors infected by *S. aureus* versus other microbes (Figures 3A–C).

### Correlations Between the Plasma Metabolic Profile and Blood Gas Analysis in Sepsis Patients

**FIGURE 4:**
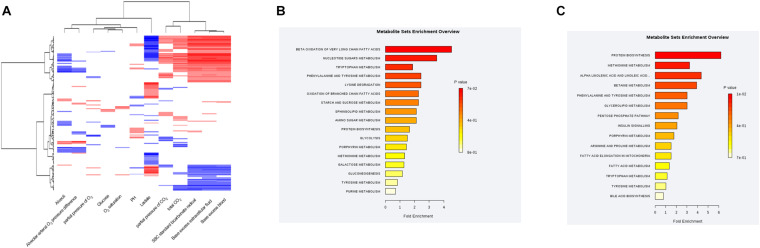
Correlations between the plasma metabolic profile and blood gas analysis in sepsis patients. **(A)** Hierarchical clustering heatmap depicting correlations between metabolomic data and blood gas indices. **(B)** Pathway enrichment analysis highlighting metabolic pathways correlated with the plasma lactate level in sepsis patients. **(C)** Pathway-enrichment analysis highlighting metabolic pathways correlated with the plasma bicarbonate level in sepsis patients.

The MS values of 514 annotated metabolites were log-transformed and normalized. Pearson correlation coefficients between the blood gas index and metabolites were then calculated, grouped by the hierarchical clustering, and presented in a heatmap form (Figure 4A). Given that lactate and bicarbonate are great prognostic values for sepsis patients, we performed enrichment analysis of those metabolites significantly related to these two clinical values. As a result, oxidation of very long chain and branched-chain fatty acids was highly enriched pathways correlated with lactate, whereas the metabolism of methionine and linoleic acids was enriched for bicarbonate. Another lipid-related pathway that warrants attention was fatty acid elongation in mitochondria (Figures 4B,C), demonstrating that the amounts of plasma lipid species are critical for the prognosis of sepsis patients.

## Discussion

The primary goal of this study is to identify appropriate metabolites, or an effective combination of metabolites, to predict the prognosis of pediatric sepsis. We performed LC-MS-based metabolomic analyses on serum samples harvested from sepsis patients and age-matched controls with detailed clinical information, and significant differences in an array of metabolites were identified during the very early stage of the disease course (from the day of admittance) between survivors and non-survivors. The data mining results indicated that most biomarkers associated with sepsis-related death were lipid molecules, especially phospholipid, and metabolites involved in energy and carbohydrate metabolism may also contribute to disease progression.

The crucial role of metabolic alterations in pathogenesis has been increasingly recognized by the field, and a vast number of research activities are devoted to the identification of metabolic biomarkers. Specifically, anti-inflammatory responses were found to be impacted by metabolism, and selective metabolites that change significantly in sepsis, SIRS, and endotoxemia were identified (Kamisoglu et al., 2015). Plenty of evidences have suggested the involvement of lipid synthesis in sepsis (Green et al., 2016), and one of the elucidated mechanisms was that lipid synthesis in macrophages can be inhibited by the deprivation of mitochondrial uncoupling protein-2 (UCP2), which was later distinguished as a potential therapeutic target for sepsis through *in vivo* and *in vitro* studies (Moon et al., 2015).

In our study, the PCA results proved that quality control samples were very well clustered, and the OPLS-DA score plot showed a perfect clustering between two examined groups, indicating effective separation and stable performance were both achieved through our analysis. Moreover, metabolic profiling revealed intriguing alterations in sepsis cases infected by different bacterial strains. Notably, different bacteria can lead to variable forms of sepsis, an urgent challenge put forward by precise management of this disease. In our analysis, we focused on the metabolites highly correlated with three major bacterial species and identified nine significantly altered metabolites, most of which belong to steroids and phospholipids. Specifically, increased levels of cholic acid, isovalerylglycine, and histidine were discovered in patients infected with *S. aureus* versus those with *P. aeruginosa* and *C. albicans*, suggesting that these metabolites may originate from immune responses in patients infected with *S. aureus*. Another possibility is that these metabolites are the secondary metabolic products of corresponding bacteria. It can be speculated that metabolic biomarkers could be utilized, together with clinical variables, to assist clinicians to distinguish bacterial species in a faster process than the traditional blood culture method.

By comparing metabolic profiles in sepsis survivors, non-survivors, and the control group, we identified 14 metabolites with elevated levels in sepsis cases versus controls, the majority of which include amino acids and carbohydrates. With respect to the differences between sepsis survivors and non-survivors, previous studies revealed that levels of metabolites related to the citric acid cycle, glycolysis, and gluconeogenic amino acids were decreased in sepsis survivors (Langley et al., 2013). In our study, we also evaluated amino acid catabolites, carnitine esters, nucleic acid catabolites, free fatty acids, glycerophosphocholines, GPC and GPE esters, glycolysis and citric acid cycle intermediates, and when sepsis survivors and non-survivors were compared, we found that two carnitine esters (palmitoylcarnitine and acetylcarnitine) and amino acids including glutamate, tyrosine, tryptophan, methionine, and proline were elevated in the non-survivor group. In addition, acetylneuraminate and N2,N2-dimethylguanosine were identified by our metabolomics platform and found increased in non-survivors; these changes were consistent with previous studies (Drobnik et al., 2003; Langley et al., 2013).

We identified a number of additional metabolites with reduced levels in sepsis survivors, implicating that other metabolic pathways may contribute to sepsis progression, especially in the Chinese population (Figure 2B). Lactate has been widely considered as a sepsis severity marker, and the level of lactate was significantly elevated in patients with worse prognosis, such as organ failure or death. We identified a series of fatty acids associated with the alteration of lactate in examined cases, including the oxidation of very long chain and branched-chain fatty acids. Therefore, the oxidative stress of fatty acids may be involved in the pathogenesis of sepsis, at least in the severe subgroup.

## Conclusion

In summary, we analyzed the global metabolomic information in a collected cohort of Chinese pediatric sepsis patients. Our study demonstrated the predictive value using the combination of metabolic biomarkers to distinguish individuals with or without sepsis, or patients with different pathogens and severities, which may lead to the development of personalized therapies against sepsis with higher accuracy.

## Data Availability Statement

The datasets generated and analyzed during the current study are available from the corresponding author on reasonable request.

## Ethics Statement

The studies involving human participants were reviewed and approved by the Ethics Committee on Human Research at Zhongshan School of Medicine, Sun Yat-sen University. Written informed consent to participate in this study was provided by the participants’ legal guardian/next of kin.

## Author Contributions

BL, Z-YO, H-YL, and CL conceived and designed the experiments. G-BL, H-RH, W-FP, BL, and CL contributed to the sample collection and metabolic analysis. G-BL, H-RH, and W-FP performed the experiments. G-BL, BL, and CL interpreted the data and wrote the manuscript. BL, Z-YO, H-YL, and CL contributed to the funding acquisition and supervision. All authors contributed to the article and approved the submitted version.

## Conflict of Interest

The authors declare that the research was conducted in the absence of any commercial or financial relationships that could be construed as a potential conflict of interest.
